# Clinical risk factors for ascites in metastatic pancreatic cancer

**DOI:** 10.1016/j.esmoop.2023.101200

**Published:** 2023-03-28

**Authors:** J.M. Berger, A. Alany, R. Puhr, L. Berchtold, A. Friedrich, B. Scheiner, G.W. Prager, A.S. Berghoff, M. Preusser, E.S. Bergen

**Affiliations:** 1 Division of Oncology, Department of Medicine I, Medical University of Vienna, Vienna, Austria; 2 Christian Doppler Laboratory for Personalized Immunotherapy, Department of Medicine I, Medical University of Vienna, Vienna, Austria; 3 Division of Gastroenterology and Hepatology, Department of Medicine III, Medical University of Vienna, Vienna, Austria

**Keywords:** ascites, metastatic pancreatic cancer, liver metastases, peritoneal carcinomatosis, systemic inflammation

## Abstract

**Background:**

Malignant ascites is common in metastatic pancreatic cancer (mPC) and its management still remains a clinical challenge. Early identification of patients at risk for ascites development may support and guide treatment decisions.

**Materials and methods:**

Data of patients treated for mPC at the Medical University of Vienna between 2010 and 2019 were collected by retrospective chart review. Ascites was defined as clinically relevant accumulation of intraperitoneal fluid diagnosed by ultrasound or computer tomography scan of the abdomen. We investigated the association between general risk factors, metastatic sites, liver function, systemic inflammation as well as portal vein obstruction (PVO) and ascites development.

**Results:**

Among 581 patients with mPC included in this study, 122 (21.0%) developed ascites after a median of 8.7 months after diagnosis of metastatic disease. The occurrence of ascites led to an 8.9-fold increased risk of death [confidence interval (CI) 7.2-11, *P* < 0.001] with a median overall survival of 1 month thereafter. Clinical risk factors for ascites were male sex [hazard ratio (HR) 1.71, CI 1.00-2.90, *P* = 0.048], peritoneal carcinomatosis (HR 6.79, CI 4.09-11.3, *P* < 0.001), liver metastases (HR 2.16, CI 1.19-3.91, *P* = 0.011), an albumin–bilirubin (ALBI) score grade 3 (HR 6.79, CI 2.11-21.8, *P* = 0.001), PVO (HR 2.28, CI 1.15-4.52, *P* = 0.019), and an elevated C-reactive protein (CRP) (HR 4.19, CI 1.58-11.1, *P* = 0.004).

**Conclusions:**

Survival after diagnosis of ascites is very limited in mPC patients. Male sex, liver and peritoneal metastases, impaired liver function, PVO, as well as systemic inflammation were identified as independent risk factors for ascites development in this uniquely large real-life patient cohort.

## Highlights

• Survival of patients with mPC is limited with 1 month after ascites diagnosis.
• Male sex, peritoneal carcinomatosis, and liver metastases are clinical risk factors for ascites.
• Systemic inflammation, impaired liver function, as well as PVO also contribute to ascites risk.

## Introduction

Ascites development is a common phenomenon in patients with malignancies, especially in tumors originating from the ovaries, colon, pancreas, and uterus.^1^^,^^2^ Treatment options for malignant ascites comprise paracentesis or permanent drainages, diuretic therapy, and treatment of the underlying malignancy.^3^, ^4^, ^5^, ^6^ However, given the high incidence of malignant ascites in certain tumor entities, treatment options did not significantly improve in recent years. One reason for this lack of effective, durable therapies may still be an insufficient knowledge on the pathophysiology of ascites development in general. Obstructed lymphatic drainage,^7^, ^8^, ^9^ increased vascular permeability caused by enhanced vascular endothelial growth factor (VEGF) levels,^10^, ^11^, ^12^ and peritoneal immunosuppression^13^^,^^14^ were reported as possible mechanisms of action. Peritoneal carcinomatosis and liver metastases have been identified as important clinical risk factors for malignant ascites so far.^15^ Additionally, systemic inflammation seems to play a crucial role in ascites development, since several inflammation parameters were linked to ascites formation in a cohort of renal cell carcinoma patients.^16^ Regardless of malignancy, portal vein obstruction (PVO) and hypoalbuminemia were also described as important risk factors for ascites.^17^^,^^18^ However, studies investigating clinical risk factors for malignant ascites so far were small and included very heterogenous patient populations with different tumor entities.

Patients with metastatic pancreatic cancer (mPC) exhibit one of the highest incidences of malignant ascites.^1^ Since these patients still face a particularly dismal prognosis compared to patients with other tumor entities, preservation of quality of life is of major interest. Considering the heavy symptomatic burden of ascites development and the associated limited prognosis, timely identification of patients at risk is of key importance especially in this distinct patient population.^16^^,^^19^ Therefore, we carried out a structured assessment of clinical risk factors for malignant ascites in a uniquely large, real-life cohort of mPC patients. Our data may support treatment decisions and may help to set up future translational research projects in this field.

## Materials and methods

### Patients

Information relating to patient demographics, case history, and survival was collected by retrospective chart review. All patients were treated according to best clinical practice and current treatment guidelines throughout their whole clinical course of disease from diagnosis of mPC onwards at our tertiary care center.^20^ This study was approved by the Ethics Committee of the Medical University of Vienna (vote number 2026 of 2021) and carried out according to the Declaration of Helsinki and its amendments.

### Study design and objectives

As primary objective we aimed to assess different clinical factors for the development of ascites. Ascites was predefined as clinically relevant accumulation of intraperitoneal fluid diagnosed either by ultrasound or computer tomography (CT) scan of the abdomen. Patients with an isolated perihepatic ascites formation were excluded from this analysis.

The association of the following risk factors with ascites development has been primarily investigated. Laboratory parameters have been grouped into ‘below normal’, ‘normal’, and ‘above normal’ according to reference intervals, which are defined as follows:

- General risk factors: age, sex
- Metastatic sites: liver, peritoneum, lung, bone
- Parameters of liver function: total protein (normal range 64-83 g/l), albumin (normal range 35-52 g/l), albumin–bilirubin (ALBI) score. The ALBI score serves as a prognostic score of liver function in patients with all stages of chronic liver disease and has been validated recently to assess prognosis in patients with hepatocellular carcinoma (HCC).^21^ Based on a calculated algorithm including serum albumin and bilirubin levels, three different grades are distinguished ranging from good (grade 1) to poor prognosis (grade 3).
- Parameters of systemic inflammation: C-reactive protein (CRP) (normal range <0.5 mg/dl), neutrophil–lymphocyte ratio (NLR), leukocyte–lymphocyte ratio (LLR), monocyte–lymphocyte ratio (MLR), platelet–lymphocyte ratio (PLR)
- PVO (by thrombosis or tumor formation) as diagnosed by radiologists based on CT scans carried out during clinical routine

### Statistical analysis

Statistical analysis was carried out using R V4.1.3. (R Development Core Team, Vienna, Austria; http://www.r-project. org). For descriptive statistics, continuous variables were presented as median and range, and categorical variables were summarized using percentages and counts. The previously described variables were investigated for their association with ascites development using Cox regression models with time-dependent covariates. Clinically relevant factors and all factors with *P* values <0.1 in the univariate models were added to a multivariate model. A two-sided *P* value of <0.05 was considered a significance threshold in the final model. Time to ascites was defined as the interval from diagnosis of mPC until diagnosis of ascites. Overall survival (OS) was defined as the interval from first diagnosis of mPC or ascites, respectively, until death or last date of follow-up. Due to the exploratory and hypothesis-generating design of the present study, no adjustment for multiple testing was applied.^22^

## Results

### Patients’ characteristics

From 824 mPC patients treated between 2010 and 2019 at the Medical University of Vienna, 306 were excluded due to incomplete data on the clinical course of disease or ascites development. Therefore, 581 patients (301 male, 280 female) with a median age of 66 years (range 35-93 years) at diagnosis of mPC were enrolled in this study. Three hundred and forty-four out of 581 patients (59.2%) presented with synchronous metastases at initial diagnosis, whereas 237 out of 581 patients (40.8%) developed metachronous metastases later throughout their course of disease. The most common metastatic sites were liver (402, 69.2%), followed by lung (154, 26.5%), peritoneum (132, 22.7%), and bone (24, 4.1%). One hundred and twenty-two out of 581 patients (21.0%) developed ascites during their course of disease after a median of 8.8 months (range 8.4-10.4 months) from diagnosis of mPC. In 12 out of 581 patients (2.1%), ascites represented the first symptom at diagnosis of mPC. Median OS from diagnosis of metastatic disease was 8.9 months (95% confidence interval (CI) 8.5-10.3 months) in the overall patient population. Patients’ characteristics are listed in Table 1.

**Table 1:** Patients’ characteristics at diagnosis of metastatic disease and diagnosis of ascites

Characteristics	At diagnosis of metastatic disease	At diagnosis of ascites
	581	122
Sex, *n* (%)		
Female	280 (48.2)	49 (40.2)
Male	301 (51.8)	73 (59.8)
Median age, years (range)	66 (35-93)	63 (36-82)
Metastatic sites, *n* (%)		
Liver	402 (69.2)	93 (76.2)
Lung	154 (26.5)	40 (32.8)
Peritoneum	133 (22.9)	76 (62.3)
Bone	24 (4.1)	9 (7.4)
Median number of metastatic sites (range)	1 (0-3)	2 (0-4)
Occurrence of metastases, *n* (%)		
Metachronous	237 (40.8)	50 (41.0)
Synchronous	344 (59.2)	72 (59.0)
Previously applied therapies, *n* (%)		
Surgery of the primary tumor	193 (39.7)	21 (22.8)
Radiation of the primary tumor	69 (12.5)	18 (15.0)
Median lines of systemic therapies (range)	2 (1-7)	2 (1-6)
Occurrence period of ascites, *n* (%)		
As first symptom of disease	12 (2.1)	
At diagnosis of metastatic disease	9 (1.5)	
During course of disease	101 (17.4)	

### Risk assessment for ascites

#### General risk factors

According to univariate analysis, a younger age [hazard ratio (HR) 0.71, CI 0.62-0.82*,* *P* < 0.001] was the only factor among general risk factors associated with ascites development. When assessed within multivariate analysis, only male sex remained independently significant (HR 1.71, CI 1.00-2.90, *P* = 0.048).

#### Metastatic sites

**Figure 1:**
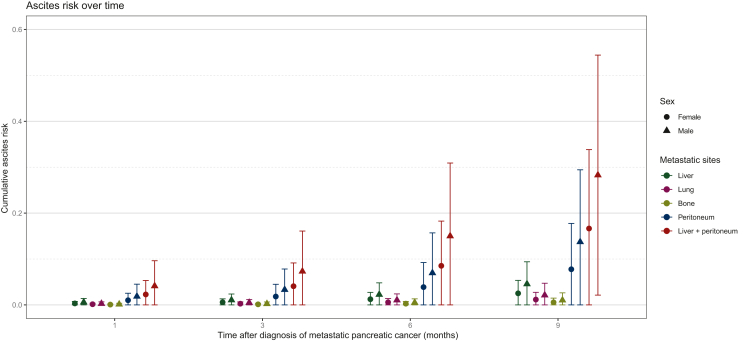
**Risk for ascites development at 1, 3, 6, and 9 months after diagnosis of mPC according to different metastatic sites [metastatic pancreatic cancer (mPC)].** The cumulative risk was calculated based on fixed parameters for female and male patients, using the mean for all continuous variables and the normal category for discrete values. For the female patients, age: 6.479, CRP: 3.7, PLR: 2211.48, LLR: 6.48, MLR: 0.55, NLR: 4.44, total protein: normal, albumin: normal, ALBI score: grade II, and portal vein structure: absent were used. For the male patients, age: 6.4, CRP: 3.78, PLR: 2211.42, LLR: 6.81, MLR: 0.64, NLR: 4.65, total protein: normal, albumin: normal, ALBI score: grade II, and portal vein structure: absent were used. ALBI score, albumin–bilirubin score; CPR, C-reactive protein; LLR, leukocyte–lymphocyte ratio; MLR, monocyte–lymphocyte ratio; NLR, neutrophil–lymphocyte ratio; PLR, platelet–lymphocyte ratio.

According to univariate analysis, peritoneal carcinomatosis (HR 5.00, CI 3.34-7.46, *P* < 0.001) and liver metastases (HR 2.80, CI 1.74-4.51, *P* < 0.001) were significantly associated with ascites development. Within multivariate analysis, the development of peritoneal carcinomatosis had the highest contemporaneous risk increase for ascites (HR 6.79, CI 4.09-11.3, *P* < 0.001) followed by liver metastases (HR 2.16, CI 1.19-3.91, *P* = 0.011). Diagnosis of liver and peritoneal metastases led to an additive risk increase. Lung and bone metastases were not associated with ascites development in univariate or in multivariate analysis (*P* > 0.05). Risk for ascites development over time according to different metastatic sites is illustrated in Figure 1.

#### Liver function

A total protein level below normal (HR 3.14, CI 1.94-5.08, *P* < 0.001), an albumin level below normal (HR 7.42, CI 4.41-12.5, *P* < 0.001), an ALBI grade of 2 (HR 5.14, CI 2.79-9.48, *P* < 0.001) or 3 (HR 29.0, CI 13.6-61.7, *P* < 0.001), and PVO (HR 5.32, CI 3.04-9.30, *P* < 0.001) were significantly associated with ascites risk in univariate analysis. Only an ALBI grade of 3 (HR 6.79, CI 2.11-21.8, *P* = 0.001) and PVO (HR 2.28, CI 1.15-4.52, *P* = 0.019) remained independently associated with ascites according to multivariate analysis.

#### Systemic inflammation

All blood cell ratios were significantly associated with ascites development within univariate analysis (NLR, LLR, MLR, PLR) as well as an elevated CRP (HR 10.9, CI 4.33-27.4, *P* < 0.001). Here, only the elevated CRP remained significant in multivariate analysis (HR 4.19, CI 1.58-11.1, *P* = 0.004).

Detailed results of the risk assessment analysis are listed in Table 2.

**Table 2:** Factors associated with ascites development in patients with mPC

	Univariate analysis		Multivariate analysis	
	HR (95% CI)	*P*	HR (95% CI)	*P*
Sex				
Female	1			
Male	1.43 (0.99-2.05)	0.054	1.71 (1.00-2.90)	**0.048**
Age at diagnosis of mPC				
(per decade increase)	0.71 (0.62-0.82)	**<0.001**	0.82 (0.66-1.03)	0.094
Metastatic sites				
Lung	1.26 (0.83-1,92)	0.278	1.10 (0.65-1.85)	0.732
Bone	1.19 (0.60-2.39)	0.618	0.48 (0.23-1.03)	0.059
Liver	2.80 (1.74-4.51)	**<0.001**	2.16 (1.19-3.91)	**0.011**
Peritoneum	5.00 (3.34-7.46)	**<0.001**	6.79 (4.09-11.3)	**<0.001**
Total protein				
Normal	1		1	
Below normal	3.14 (1.94-5.08)	**<0.001**	1.74 (0.94-3.20)	0.076
Albumin				
Normal	1		1	
Below normal	7.42 (4.41-12.5)	**<0.001**	1.88 (0.79-4.51)	0.156
ALBI score				
Grade 1	1		1	
Grade 2	5.14 (2.79-9.48)	**<0.001**	1.58 (0.64-3.93)	0.322
Grade 3	29.0 (13.6-61.7)	**<0.001**	6.79 (2.11-21.8)	**0.001**
CRP				
Normal	1		1	
Above normal	10.9 (4.33-27.4)	**<0.001**	4.19 (1.58-11.1)	**0.004**
NLR				
(per unit increase)	1.04 (1.02-1.05)	**<0.001**	0.97 (0.94-1.01)	0.136
LLR				
(per unit increase)	1.03 (1.02-1.04)	**<0.001**	1.02 (0.99-1.06)	0.260
MLR				
(per unit increase)	1.51 (1.24-1.84)	**<0.001**	1.25 (0.86-1.80)	0.244
PLR				
(per 100 unit increase)	1.25 (1.14-1.36)	**<0.001**	1.10 (0.99-1.23)	0.086
Portal vein obstruction				
Absent	1		1	
Present	5.32 (3.04-9.30)	**<0.001**	2.28 (1.15-4.52)	**0.019**

Univariate and multivariate analysis stratified by Cox regression models with time-dependent covariates.

Bold values indicate statistical significance.

ALBI score, albumin–bilirubin score; CI, confidence interval; CRP, C-reactive protein; HR, hazard ratio; LLR, leukocyte–lymphocyte ratio; MLR, monocyte–lymphocyte ratio; mPC, metastatic pancreatic cancer; NLR, neutrophil–lymphocyte ratio; PLR, platelet–lymphocyte ratio.

### Clinical presentation, management, and outcome of patients with ascites

At ascites diagnosis, patients had undergone a median of 2 therapy lines (range 0-5) and had a median Eastern Cooperative Oncology Group performance score of 2 (range 0-4). One hundred and eighteen out of 122 (96.7%) patients were symptomatic at diagnosis of ascites. Main symptoms of patients were dyspnea in 89 out of 122 patients (73.0%) and pain in 85 out of 122 patients (69.7%). The most frequently applied treatment for ascites was serial paracentesis in 110 out of 122 patients (90.2%) with a median of 1 re-puncture (range 0-8). The median volume retrieved at the first paracentesis was 5 l (range 0-11). A permanent drainage was implanted in 53 out of 122 patients (43.4%). Median OS from diagnosis of ascites was 1 month (range 0.7-1.4). The occurrence of ascites was associated with an 8.9-fold increase in risk for death (CI 7.2-11, *P* < 0.001).

## Discussion

The aim of the present study was to identify clinical risk factors for the development of ascites in a large cohort of mPC patients. This seems crucial since outcome of mPC patients in general remains dismal and development of ascites contributes substantially to the high symptom burden this distinct patient cohort is facing.

Median OS in the present cohort of mPC patients was 8 months and therefore comparable with outcome data of larger, prospective trials in this setting.^23^^,^^24^ Incidence of ascites was 21.0%, which is also well in line with previous mPC cohorts emphasizing the relevance this symptom has in daily clinical practice.^25^, ^26^, ^27^, ^28^ Compared to other intra-abdominal tumor entities like gastric cancer, ascites in mPC has a higher incidence.^16^^,^^29^ Ascites thereby does not only cause a considerable symptomatic burden for patients, but also increases the risk of death.^16^^,^^19^ The median OS after ascites diagnosis in this study was only 1 month, which is considerably shorter compared to patients with other tumor entities developing ascites.^1^ However, since previous studies on ascites mostly comprise patients with ovarian cancer, direct comparisons between patient populations cannot be drawn. Although ascites represents an end-stage event in mPC, it often occurs as one of the first symptoms in ovarian cancer and may be present even at a curative stage.^30^

This study is the first to identify men to be at independently higher risk for ascites development. Results from mixed cancer cohorts rather suggest that women are at higher risk for ascites as gynecologic malignancies most frequently cause ascites development.^1^ However, in studies comprising pancreatic cancer or renal cell carcinoma patients, no statistically significant difference with regard to sex could be observed so far.^16^^,^^28^ Interestingly, a higher age seemed to be protective against ascites development at least according to univariate analysis in our study. A higher tumor burden and a more aggressive disease in younger patients may therefore serve as explanation. Moreover, younger patients may live longer and therefore exhibit a longer period of time when ascites can be diagnosed.

The presence of liver metastases and peritoneal carcinomatosis was previously reported to be associated with ascites development.^15^^,^^16^ Here, we were able to identify both metastatic sites as independent factors for ascites formation with an additive risk increase in patients with these sites co-occurring. Liver metastases most likely increase the risk for ascites causing portal hypertension and limiting functional liver reserve, whereas peritoneal metastases are known to cause lymphatic vessel obstruction and increase vascular permeability.^7^, ^8^, ^9^, ^10^, ^11^, ^12^ Portal hypertension has been previously described to be closely related to ascites formation, not only in patients with liver cirrhosis but also in patients with HCC and liver metastases.^31^^,^^32^ In patients with mPC, PVO may be either caused by tumor infiltration or thrombosis of the portal vein, resulting in portal hypertension and impaired liver function.^33^^,^^34^ Within the present study, PVO acted as an independent risk factor for ascites. Therefore, early anticoagulation at diagnosis of portal vein thrombosis (PVT) may be considered to counteract ascites development even though data addressing the treatment of malignant PVT are limited. Independent of PVO, parameters of liver function were found to be associated with ascites formation. In patients with liver cirrhosis as well as HCC, the ALBI score was shown to determine the liver functional reserve and predict survival outcomes.^18^^,^^35^ Reduced albumin production and an associated hypoosmotic state most likely contribute to the pathophysiology of ascites also in mPC patients.^18^ This implies that the liver function should be monitored in these patients and albumin substitution may be considered in case of low albumin levels. Further, prospective interventional studies on liver function substitution as well as anticoagulation for PVT may contribute to delay the onset of ascites development. Here, the optimal timepoint and most effective intervention are to be determined.

Another novel finding is the independent association between systemic inflammation and ascites development. According to previous data, serum CRP was shown to be elevated in patients with malignant ascites compared to patients with benign ascites.^36^ However, there are only a few reports linking parameters of systemic inflammation like PLR with ascites development, but none in patients with mPC.^16^ As this is the first study showing an association between systemic inflammation and ascites development, additional data are highly warranted to identify potential novel targets for immunomodulatory treatment of malignant ascites. This seems especially important since no systemic therapy approaches for ascites are available so far, chemotherapy often is not feasible considering the impaired performance status of these patients, and diuretics seem not sufficient for ascites control in the majority of patients as indicated by the high rate of serial paracentesis of 90% in our ascites patients. Also, the monoclonal VEGF receptor antibody bevacizumab did not result in a longer puncture-free survival in patients with gastrointestinal malignancies, when applied intraperitoneally as recently reported by a phase II prospective trial.^37^ This novel finding may therefore influence research efforts to identify additional targeted treatment options. Given the limited prognosis after ascites diagnosis, however, further research is highly needed to investigate if patients benefit from a continuation of antitumoral therapy at this stage.

As this study is a retrospective assessment of ascites risk factors, it is naturally limited by its design. Also, there is some heterogeneity within the study population resulting from the long observational period and the inclusion of patients with synchronous and metachronous metastases. A more detailed assessment of the underlying cause of ascites would have been of interest, but cytological work-up was only available in a few patients. However, this is the first structured analysis of risk factors for ascites development in patients with mPC based on a uniquely large cohort of more than 500 individuals.

In conclusion, ascites in patients with mPC may be caused by a multitude of clinical factors. Their assessment together with a multidisciplinary management is important to reduce the high symptomatic burden associated with ascites in order to preserve quality of life in this patient cohort.
